# The B.M.A. and the Medical Guild

**Published:** 1913-08-23

**Authors:** 


### THE NATIONAL MEDICAL GUILD.

President: CHAS. BUTTAR, M.A., M.D., B.C.

Gen. Sec.: P. C. RAIMENT, M.R.C.S., L.R.C.P.

Offices: 34 Villiers Street, Strand, London, W.C.

*[On this and the following page have appeared during the last few weeks communications from the supporters and upholders of the National Medical Guild. Last week we published from the same source a general criticism and account of "Professional Mental Instability and its Dangers." This week we publish a reply from Dr. E. Rowland Fothergill, a member of the British Medical Association, in reply to last week's propaganda, and also answers to Dr. Fothergill's seven questions from the representatives of the Guild who supplied the previous article.*—Ed. The Hospital.]

### THE B.M.A. AND THE MEDICAL GUILD.

### THE B.M.A. AND STATE SICKNESS INSURANCE.

We have received the following statement signed E. Rowland Fothergill, M.B., B.S., Brighton:—

In your issue for August 16 there appears under this heading a criticism of a scheme proposed for the adoption of the British Medical Association for forming a voluntary fund, to be used for the prevention and cure, so far as is possible and practicable, of the ills the medical profession collectively and individually are suffering from. In many particulars the statements made are inaccurate, and the concluding deduction that the scheme costs 13s. 4d. in order to collect £1 is a misstatement of fact. For the moment I am not concerned to defend this proposed scheme beyond stating that the suggested reserve of £13,000 per annum is only one of many of the objects for which the scheme is advocated. The balance would not be swallowed up in administration expenses.

Questions for the Medical Guild.

As it is advocated that by means of a Trade Union one may reasonably hope for the cure-all and mend-all of the ills the medical profession suffers from, may I beg the use of your Journal in order to ask the President of the National Medical Guild a few questions, the answers to which will no doubt prove of great utility to your numerous readers?

1. Workmen, for whom a Trade Union proves a powerful weapon, work in groups or large bodies. Agricultural labourers are an exception; especially so are medical practitioners, who work entirely as individuals. How, then, is it proposed by means of a Trade Union to interfere successfully with the practice of a doctor who is a member so as to render it incumbent on him to obey the decisions of his Union, if he declines to do so?

2. Collective bargaining is one of the fundamental necessities in any Trade Union. How is it proposed to compel a medical member or members to refrain from making their own bargain with a public or private authority, if determined to do so?

3. The maintenance of a Standard Rate is vital to the success of any Trade Union. How is it proposed to compel a medical member or members to refrain from selling their services at a lower rate than the standard adopted, if determined to do so?

4. A Trade Union would be impotent and a misnomer if not provided with strike, lock-out, or out-of-work funds. How is it proposed to compel a medical member or members to pay their levies when they know that such levy cannot be enforced in a court of law, if they decline to do so?

5. The funds of a Trade Union can be secured against appropriation in a case of "conspiracy" or libel by the Union; but not so the private property of the directors or officials acting in the official capacity. How is it proposed to meet this difficulty in the case of a *medical* Trade Union?

6. A member of a Trade Union can resign at any time, even after a levy notice has been received. How is it proposed to prevent such resignations being sent in wholesale directly a levy is made, seeing that no court of law can enforce a fine incurred by so doing?

If the answer to any one or more of these questions is "turn him out of the Union," then another question arises:—

7. How will turning a doctor or a number of doctors out of a Trade Union harm him or them? Will it not create public sympathy in *his* or *their* favour and thereby assist in an increase of practice and money earned?

These are a few questions to begin with. As one who has had during the past twelve years a close and intimate knowledge of the medical profession behind the scenes, I am convinced of two things: that no Trade Union for medical practitioners (1) can make a silk purse out of a sow's ear, or (2) get blood out of a stone. Then why form one?

If it should prove any assistance in answering these questions, I am willing to concede that *before* troubles commence or before the directors of the Trade Union issue orders to fight, or to pay out a levy, or to resign certain appointments held, that there are 20,000 medical practitioners out of a possible 32,000 in the Union.

### THE NATIONAL MEDICAL GUILD'S REPLY.

Dr. Rowland Fothergill's letter betrays that confusion of intellect that we spoke of last week as dominating the profession at the present moment. In his opening sentence it will be seen that he charges the writers with making a misstatement of fact, which in the next paragraph he admits is the truth. *Litera scripta manet.* We stand by every word that we said on August 16, and we would ask Dr. Fothergill if it is not a fact that the imaginary balance sheet of the fund in question only shows a reserve of £13,000 out of a total annual sum to be collected of £50,000? If this is true, where is the misstatement when we say the scheme provides for an expenditure of 13s. 4d. to collect each sovereign? But, says Dr. Fothergill, "the reserve is only one of the many objects for which the scheme is advocated." Quite so. The other objects apparently are the offices, staff, committees, and secretaries of the B.M.A., together with their legal advisers and general helpers. All this machinery each year swallows the 13s. 4d., and can reasonably be called administration. If this is not so we know not the meaning of the words administrative expenses.

Having relieved his mind of the mistaken statements referred to above, Dr. Fothergill proceeds to elaborate a series of questions that he addresses to our President. He premises that a Trade Union is the " cure-all and mend-all " of the ills of the profession. Wrong again, Dr. Fothergill. We have never urged that Unionism *per se* is the panacea for all our woes, but what we have said, and what we reiterate, is that the profession will be in a stronger position if it takes advantage of the privileges afforded to Trade Unions.

### The Guild's Answers to the Seven Questions.

Now to answer his questions. They are not particularly new and seem to cover the ground of inquiries for which the British Medical Association has sought the answers in vain; and it has sought them in vain because the Council have never wished, or never been able, to offer any lead to the profession, but have continually sent down to Divisions a mass of ill-digested problems with no guide to them how to deal with them. These have been discussed at representative meetings, and again referred to the Council, and so a vicious circle has been established from which there is no escape.

1. In answer to the first question we have to point out that the agricultural labourer is not a very good example to choose, as the Labourers' Union is now rapidly going ahead and the agricultural labourer is beginning to be organised. Further, as regards the interference with practice: What has the B.M.A. been doing all these years with its warning notices and its endeavour to prevent men taking appointments at unfair rates? Is not this interference with practice? and what the B.M.A. has done with the limited powers at its disposal cannot a Trade Union do more effectually? Further, has Dr. Fothergill never heard of the New South Wales Branch of the B.M.A.?

2. With regard to the second question, we can only reiterate what we have just said. Men cannot be prevented from becoming blacklegs and the Association is continually trying to impose collective bargaining on the profession. How much more successful it could be if it possessed Trade Union powers is a matter for debate. We hold that it could do better. Dr. Fothergill probably holds otherwise, and we shall be pleased to debate the matter further with him.

3. The third question appears to be a repetition of the two previous ones and the same remarks apply. Everyone has recognised that this is the crux of the whole question before the profession. What we urge is that the legal safeguards of a Trade Union give greater facilities for bringing pressure to bear on the recalcitrant member.

### The Problem of Levies.

4. The next query deals with levies. We will answer it by asking a question in return. What guarantee has the B.M.A. that anyone will subscribe to the proposed defence fund? There is no legal liability. What would make the profession subscribe is a feeling of confidence in the work of the body to which they belong. Why do we all pay to the Medical Defence Union? Why do the non-panel men join the Guild? Because we fought the case of Heard *v.* Pickthorne (refused by the B.M.A., be it noted) in their interests, and established once for all a very valuable right for them. So we hope to go on assisting the profession at every point and attracting them to join the Trade Union. At any rate we have confidence that the profession will be more likely to subscribe £2, the *whole* of which will be placed to reserve for the benefit of the profession, rather than give a five-pound note, of which £3 6s. 8d. is hypothecated in advance to expenses.

5. In the fifth question Dr. Fothergill has rather allowed his bias against Trade Unions to run away with his legal judgment. It is not yet absolutely clear that the private means of an official are liable to attachment. The point is doubtful, the Guild knows, but we are prepared to take the risk, and are willing to allow the Government to proceed against us individually if they should have any cause, or should think fit to do so. Of one thing we are certain—the sympathy and help of the profession and the general public would be with us to such an extent as to put the matter out of the realms of practical politics.

### Members who Resign.

6. Sixthly, how can we prevent the members resigning? We cannot, but we ask in all seriousness would any man join if he were not prepared to meet his levies? Further, we have always preached the necessity of money for defence, and if the profession is not prepared to put up that money then neither the British Medical Association, the National Medical Guild, or any other body can do anything, and the profession must wander along as best it may. We believe that they can and will subscribe to any reasonable fund that will provide, as the Medical Guild can and will, for their security.

### The Expulsion of Members.

Lastly, the justification for expulsion of a member or members would rest upon the justice of the cause. No man cares to be expelled *publicly* from his professional organisation, and we think that at the height of the enthusiasm of last year no man would have received very much encouragement from the general public if it had been necessary to remove him from the British Medical Association.

### The Folly of Fear and its Fruits.

In fact, the whole of Dr. Fothergill's questions have their origin in that fear which characterised the effete leaders of the profession last year; men incapable of taking any wide view of the situation; incapable of acting in the spirit of the resolutions passed at one representative meeting after another. The majority of men need strong leaders. In a Trade Union they may find them, in the British Medical Association they have failed to do so.

